# 362. Safety and Immunogenicity of a Variant-adapted Bivalent (Original/Omicron BA.4/BA.5) BNT162b2 COVID-19 Vaccine Given as a Booster (Dose 4) to Toddlers and Children 6 Months to < 5 Years of Age Who Previously Received Original BNT162b2 as a 3-Dose Primary Series

**DOI:** 10.1093/ofid/ofad500.432

**Published:** 2023-11-27

**Authors:** Lawrence Sher, Charu Sabharwal, Nicholas Kitchin, Justice Kofi Boakya-Appiah, Xia Xu, Emmanuel Walter, Yvonne A Maldonado, Flor M Munoz, Janet A Englund, Kawsar R Talaat, Shelly Senders, Satoshi Kamidani, Elizabeth Barnett, Grant C Paulsen, Lisa Moyer, Vrunda Parikh, Hua Ma, Xingbin Wang, Kenneth Koury, Annaliesa S Anderson, Kena A Swanson, Alejandra C Gurtman, William C Gruber

**Affiliations:** Peninsula Research Associates, Rolling Hills Estates, California; Pfizer Inc, Pearl River, New York; Pfizer Inc, Pearl River, New York; Vaccine Research and Development, Pfizer Ltd, Hurley, UK, London, England, United Kingdom; Pfizer Inc, Pearl River, New York; Duke Human Vaccine Institute, Durham, North Carolina; Stanford University, Stanford, California; Baylor College of Medicine, Houston, TX; Seattle Children’s Hospital, Seattle, Washington; Johns Hopkins Bloomberg School of Public Health, Baltimore, MD; Senders Pediatrics, Cleveland, Ohio, United States, Euclid, Ohio; Emory University School of Medicine and Children's Healthcare of Atlanta, Atlanta, Georgia; Boston Medical Center, Boston, Massachusetts; Cincinnati Children's Hospital Medical Center, Cincinnati, Ohio; Pfizer Inc, Pearl River, New York; Vaccine Research and Development, Pfizer Inc, Pearl River, Pennsylvania; Vaccine Research and Development, Pfizer Inc, Pearl River, Pennsylvania; Vaccine Research and Development, Pfizer Inc, Pearl River, Pennsylvania; Pfizer, Pearl River, NY; Pfizer, Pearl River, NY; Pfizer, Pearl River, NY; Pfizer, Pearl River, NY; Pfizer, Pearl River, NY

## Abstract

**Background:**

A variant-adapted bivalent BNT162b2 vaccine (bivalent BNT162b2) comprised of original SARS-CoV-2 and Omicron BA.4/BA.5 spike proteins was developed to improve protection against SARS-CoV-2 variants. Bivalent BNT162b2 is authorized by the US FDA from 6 months (mo) old; for children 6 mo to < 5 years (y), it is authorized as a 3-dose primary series and as a fourth dose (first booster).

**Methods:**

This substudy is part of a phase 1/2/3 master study (NCT05543616) investigating safety and immunogenicity of bivalent BNT162b2. The group reported here is open label and evaluates a fourth dose (first booster) with bivalent BNT162b2 3 μg in children 6 mo to < 5 y who previously received 3 original BNT162b2 3 μg doses. Reactogenicity (7 day), and 1 mo safety and immunogenicity for a subset of 60 participants were assessed. Descriptive immunogenicity endpoints included SARS-CoV-2 Omicron BA.4/BA.5 and ancestral strain neutralization responses post dose 4. A comparator group for immunogenicity included 60 participants, matched by age and SARS-CoV-2 infection status, from the initial pediatric study (NCT04816643) who received 3 original BNT162b2 3 μg doses without a booster.

**Results:**

Of children 6 mo to < 5 y old (6 mo to < 2 y, n = 24; 2 y to < 5 y, n = 36) who received bivalent BNT162b2 as a fourth dose, 50% were male, 58% White, 5% Black, 15% Asian, 22% multiracial, 25% Hispanic/Latino; 28.3% had evidence of past SARS-CoV-2 infection. Median time from dose 3 of original BNT162b2 to bivalent BNT162b2 was 6.5 mo. Bivalent BNT162b2 was generally well tolerated with mostly mild to moderate reactogenicity and minimal fever (**Figure**), few reported adverse events (AEs), no serious AEs, and no new safety signals identified. Bivalent BNT162b2 elicited higher neutralizing titers against Omicron BA.4/BA.5 and similar titers against ancestral strain 1 mo post dose 4 compared with original BNT162b2 1 mo post dose 3 (**Table**). Robust immune responses were observed regardless of previous SARS-CoV-2 infection status.

**Figure:**
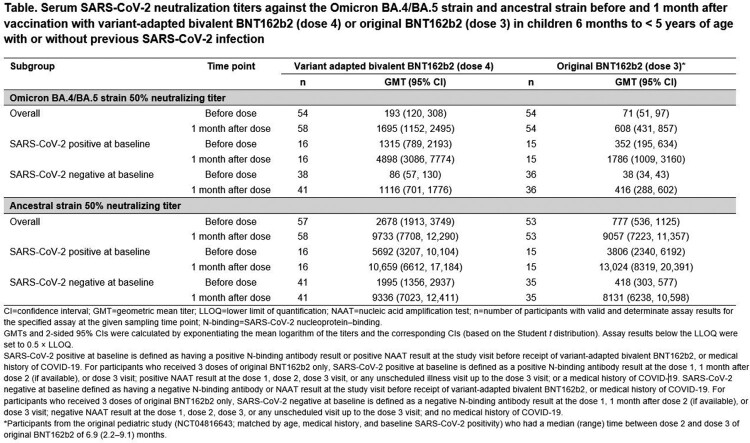

**Figure:**
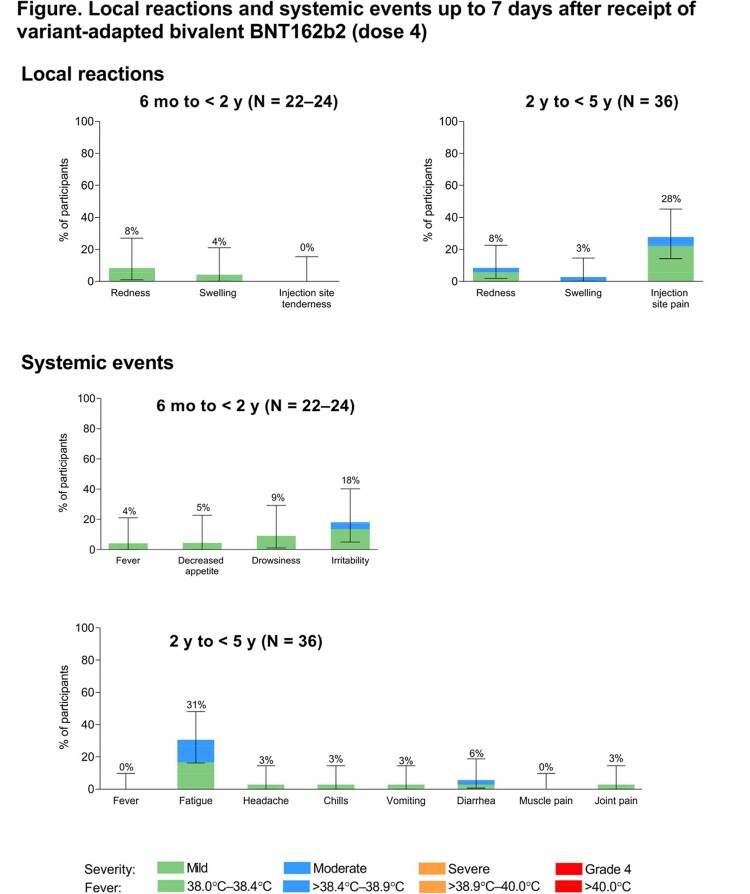

**Conclusion:**

A booster (dose 4) of bivalent BNT162b2 had a similar safety profile to original BNT162b2 and induced robust Omicron BA.4/BA.5 and ancestral strain neutralizing titers in children 6 mo to < 5 y. These descriptive data reinforce the importance of a variant-adapted bivalent COVID-19 booster dose in this age group.

**Disclosures:**

**Lawrence Sher, MD**, Pfizer Inc: Clinical Investigator **Charu Sabharwal, MD, MPH**, Pfizer Inc: Employee|Pfizer Inc: Stocks/Bonds **Nicholas Kitchin, MD**, Pfizer Inc: Employee|Pfizer Inc: Stocks/Bonds **Justice Kofi Boakya-Appiah, MD**, Pfizer Inc: Employee|Pfizer Inc: Stocks/Bonds **Xia Xu, PhD**, Pfizer Inc: Employee|Pfizer Inc: Stocks/Bonds **Emmanuel Walter, MD**, Clinetic: Clinical Investigator|Iliad Biotechnologies: Advisor/Consultant|Moderna: Clinical Investigator|Najit Technologies: Clinical Investigator|Pfizer Inc: Clinical Investigator|Sequiris: Clinical Investigator|Vaxcyte: Advisor/Consultant **Yvonne A. Maldonado, MD**, Pfizer: Grant/Research Support|Pfizer: Site Investigator, DSMB member **Flor M. Munoz, MD, MSc**, CDC respiratory virus surveillance: Grant/Research Support|Gilead: Grant/Research Support|Moderna, sanofi, aztra zeneca, Merck, GSK: Advisor/Consultant|NIH: DSMB|NIH COVID-19 vaccines in pregnancy: Grant/Research Support|Pfizer Pediatric COVID-19 vaccines: Grant/Research Support|Pfizer, Dynavax, Monderna, Meissa, NIH: DSMB **Janet A. Englund, MD**, Ark Biopharma: Advisor/Consultant|AstraZeneca: Advisor/Consultant|AstraZeneca: Grant/Research Support|GlaxoSmithKline: Grant/Research Support|Meissa Vaccines: Advisor/Consultant|Merck: Grant/Research Support|Moderna: Advisor/Consultant|Moderna: Grant/Research Support|Pfizer: Advisor/Consultant|Pfizer: Grant/Research Support|Sanofi Pasteur: Advisor/Consultant **Kawsar R. Talaat, MD**, Intralytix: Advisor/Consultant|Merck: Advisor/Consultant|NIAID: DSMB|Pfizer: Grant/Research Support|Pfizer: Pfizer contract with institution|Sanofi: Grant/Research Support|Takeda: Advisor/Consultant **Satoshi Kamidani, MD**, CDC: Grant/Research Support|Emergent BioSolutions: Grant/Research Support|NIH: Grant/Research Support|Pfizer Inc: Grant/Research Support **Grant C. Paulsen, MD**, Moderna: Grant/Research Support|Pfizer: Grant/Research Support **Lisa Moyer, BS**, Pfizer: Employee|Pfizer: Stocks/Bonds **Vrunda Parikh, PharmD**, Pfizer: Employee|Pfizer: Stocks/Bonds **Hua Ma, PhD**, Pfizer: Employee|Pfizer: Stocks/Bonds **Xingbin Wang, PhD**, Pfizer: Employee|Pfizer: Stocks/Bonds **Kenneth Koury, PhD**, Pfizer: Employee|Pfizer: Stocks/Bonds **Annaliesa S. Anderson, PhD**, Pfizer: Employee|Pfizer: Stocks/Bonds **Kena A. Swanson, Ph.D.**, Pfizer: Employee|Pfizer: Stocks/Bonds **Alejandra C. Gurtman, M.D**, Pfizer: Employee|Pfizer: Stocks/Bonds **William C. Gruber, MD**, Pfizer, Inc.: Employee|Pfizer, Inc.: Stocks/Bonds

